# Immunological impact of an additional early measles vaccine in Gambian children: Responses to a boost at 3 years

**DOI:** 10.1016/j.vaccine.2012.01.083

**Published:** 2012-03-28

**Authors:** Jainaba Njie-Jobe, Samuel Nyamweya, David J.C. Miles, Marianne van der Sande, Syed Zaman, Ebrima Touray, Safayet Hossin, Jane Adetifa, Melba Palmero, Sarah Burl, David Jeffries, Sarah Rowland-Jones, Katie Flanagan, Assan Jaye, Hilton Whittle

**Affiliations:** a Medical Research Council Laboratories, P.O. Box 273, Banjul, Gambia; b National Institute of Health and Environment (RIVM), Centre for Infectious Disease Control Netherlands, Bilthoven, Netherlands; c Julius Centre for Health Sciences, University of Utrecht Medical Centre, Utrecht, Netherlands; d Health Protection Agency, Centre for Infection, Colindale, London, NW9 5EQ, UK; e Adventist Community Services, Adventist Health Ministries, Anchorage, AK 99507, USA; f Department of Paediatrics, Imperial College, London, W2 1PG, UK; g Weatherall Institute of Molecular Medicine, University of Oxford, Oxford, OX3 9DS, UK

**Keywords:** Measles vaccine, Two-dose schedule, Booster dose, Antibody, Cellular immunity

## Abstract

**Background:**

Measles vaccine in early infancy followed by a dose at 9 months of age protects against measles and enhances child survival through non-specific effects. Little is known of immune responses in the short or long term after booster doses.

**Methods:**

Infants were randomized to receive measles vaccine at 9 months of age (group 1) or 4 and 9 months of age (group 2). Both groups received a boost at 36 months of age. T-cell effector and memory responses using IFN-γ ELIspot and cytokine assays and antibody titres using a haemagglutination-inhibition assay were compared at various times.

**Results:**

Vaccination at 4 months of age elicited antibody and CD4 T-cell mediated immune responses .Two weeks after vaccination at 9 months of age group 2 had much higher antibody titres than group1 infants; cell-mediated effector responses were similar. At 36 months of age group 2 antibody titres exceeded protective levels but were 4-fold lower than group 1; effector and cytokine responses were similar. Re-vaccination resulted in similar rapid and high antibody titres in both groups (median 512); cellular immunity changed little. At 48 months of age group 2 antibody concentrations remained well above protective levels though 2-fold lower than group 1; T-cell memory was readily detectable and similar in both groups.

**Conclusions:**

An additional early measles vaccine given to children at 4 months of age induced a predominant CD4 T-cell response at 9 months and rapid development of high antibody concentrations after booster doses. However, antibody decayed faster in these children than in the group given primary vaccination at 9 months of age. Cellular responses after 9 months were generally insignificantly different.

## Highlights

► Gambian infants were given one or two doses of measles vaccine. ► The kinetics of the immune response was compared after a boost. ► Antibody responses were equally rapid and high. ► Cell mediated responses were insignificantly different. ► Antibody concentrations decayed quicker in the two dose group.

## 1 Introduction

In Africa the timing of the first dose of measles vaccine at 9 months of age is an uneasy compromise designed to minimize interference from maternal antibody and to provide protection for the maximum number of infants [1]. Unfortunately some children of mothers who have been vaccinated rather than naturally infected with measles lose maternal antibody long before this age. As vaccine coverage has increased more infants have become susceptible to measles at a younger age [2].

Two strategies have been proposed to overcome this problem. Recently expensive mass vaccination campaigns have been deployed to increase coverage and provide an opportunity for two or more doses of measles vaccine. Thus herd immunity has been enhanced co-incidentally protecting unimmunized infants [3].

Another strategy is to immunize children twice in infancy. Such a regimen when used in Guinea–Bissau resulted in high coverage, high antibody concentrations, excellent protection against measles [4,5] and enhanced child survival through non-specific effects by 30% [6]. These studies used the Edmonston-Zagreb (E-Z) strain of measles vaccine which produces higher antibody concentrations than other measles vaccines when maternal antibody is present [7] or when used to boost antibody [8].

Research in the U.S.A. has shown that cell mediated responses to measles vaccine given to children at 6 months of age were similar to those in children vaccinated at 9 or 12 months of age but antibody responses were diminished by maternal antibody. However 6 months after a boost at 12 months of age protective levels of antibody were achieved in 86% of the youngest children while T-cell proliferative responses changed little in any of the age groups [9]. Vaccine effectiveness of an early two dose schedule during a large measles epidemic in Florida was 99% [10].

Despite the widespread use of repeated mass measles re-vaccination in Sub Saharan Africa little is known of the resulting immune responses, their short term kinetics or their duration in African children. Thus we compared cell mediated and antibody responses in Gambian infants at various time points after one or two doses of measles vaccine and after a booster dose at 3 years of age.

## 2 Methods

### 2.1 Subjects

This study took place in Sukuta, a peri-urban village in The Gambia. The cohort of children, criteria for selection and site have been described elsewhere [11].

**Fig. 1:**
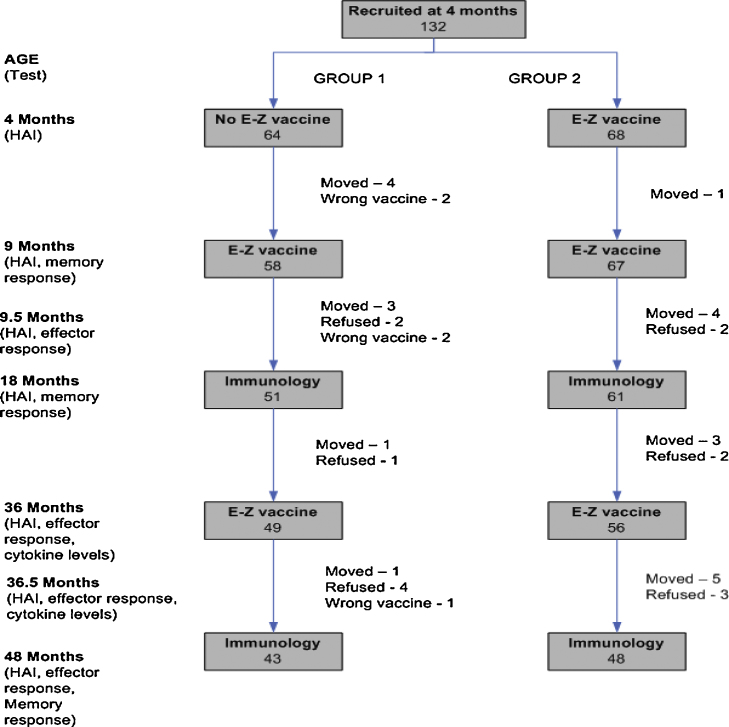
The design of the study, the number of children vaccinated and bled at each time point and the immunological tests undertaken are shown in this figure.

Fig. 1 shows the design of the study, the number of children at each time point and the various immunological tests undertaken.

The studies were approved by the local MRC Scientific Committee and by the Joint Gambian Government/MRC Ethics Committee.

### 2.2 Vaccines, vaccine schedules and follow-up

At 4 months of age infants were allocated using random numbers to receive either no measles vaccine (group 1) or a standard dose of E-Z measles vaccine (group 2) consisting of 3700 plaque forming units (Serum Institute of India, Pune) given intramuscularly in the left upper arm. EPI vaccines including a 3rd dose of Hepatitis B, DTP and Hib vaccines and a 4th dose of oral polio vaccine were also given. At 9 months of age in addition to yellow fever vaccine given in the other arm group 1 received their first dose of measles vaccine and group 2 their second dose. At 36 months of age of age both groups received another dose of measles vaccine. In order to avoid frequent venous bleeds children were also randomised either to be tested for memory responses at 9 months of age or effector responses at 9.5 months of age (details not shown). To assess safety home visits were conducted thrice in the two weeks following measles vaccination at 4 and 9 months.

### 2.3 Laboratory methods

*Serology*: Measles haemagglutination-inhibiting (HAI) antibody which correlates strongly with neutralizing antibody [12] and is quicker and easier to assay than the plaque reduction neutralization assay, was measured by use of *Chlorocebus Aethiops* red blood cells (Barbados Primate Research Centre) as previously described [13]. The sensitivity of the assay was 15.6 mIU/ml and the minimum detection level 31.2 mIU/ml. Results were expressed as log_2_ units or as reciprocal titres. We defined the protective level of HAI measles antibody as a titre of log_2_ ≥ 3 which equates to 125 mIU [12].

Ex vivo *measles effector cell assays:* After separation of blood on Lymphoprep PBMC were used in the ex vivo interferon-gamma (IFN-γ) ELIspot assay as previously described [14]. The cells were infected for 2 h with Edmonston (E-D) wild type measles virus or E-Z measles vaccine virus which had been grown for 3 days on a culture of Vero cells in RPMI/10% Foetal Calf Serum (R10F). The multiplicity of infection was 0.1 and 1.0 for the two strains respectively. The infected cells were then washed and plated in duplicate at 10^5^ cells/well in R10 with 10% AB serum (R10AB, Sigma). Control PBMC were mock infected with R10F harvested after culture of uninfected Vero cells for 3 days.

In addition duplicate wells containing 10^5^ PBMCs were also stimulated with a pool of overlapping 20-mer measles fusion peptides (NMI Peptides) dissolved in normal saline and 0.4% DMSO and used at a final concentration of 2 μg/ml in R10AB. Control cells were incubated in medium containing 0.02% DMSO which was the same concentration as that in the test wells. Phytohemagglutinin (5 μg/ml) was used as a positive control.

Spots were counted using the AID ELIspot plate reader (Autoimmune Diagnostika). The mean number of spots in the duplicate wells of the negative control was subtracted from the mean spot count in the positive wells; an assay with a control value of ≥50 spots per well was regarded as invalid.

*Measles memory cell assays:* As described previously 10^6^ PBMC were cultured for 10 days in R10AB with 10^5^ UV irradiated PBMC infected with measles virus [15] or with pooled measles nucleoprotein or fusion peptides as described above. Controls consisted of PBMC mock infected with Vero cell medium and treated in the same way as above.

*Intracellular cytokine staining (ICS):* Following stimulation, cells were permeabilised and stained for flow cytometry analysis as previously described [13]. The staining panel used at 9 and 9.5 months was anti-CD8 FITC, anti-CD4 PE, anti-CD69 PerCP and anti-IFN-γ APC. At 18 months, the panel was anti-IFN-γ FITC, anti-CD4 PE, anti-CD8 PerCP and anti-IL-2 APC. All antibodies were supplied by BD Biosciences.

*Cytokines in plasma or supernatants:* Plasma was frozen at −40° C until assayed using the Bio-Plex 200 Suspension Array system (Bio-Rad) according to the manufacturer's instructions.

*FOXP3 mRNA expression:* RNA was extracted from whole blood collected in Paxgene tubes (PreAnalytix, QIAGEN) and frozen at −40° C until RNA extracted. RNA was reverse transcribed into cDNA using 1 μM oligo-dT (Sigma-Genosys) and 10 units of ribonuclease inhibitor (Invitrogen). Gene expression was measured by real time PCR (RT-PCR) using the Corbet Research Rotor gene 6000 with the QuantiTech SYBR Green kit (QIAGEN). The FOXP3 sequences used were: forward primer 5′-ACCTGGAAGAACGCCAT and reverse primer 5′-TGTTCGTCCATCCTCCTTTC both at a final concentration of 0.4 μM. FOXP3 copy numbers were expressed in relation to human acidic ribosomal protein (HuPO), the house keeping gene.

The standards were prepared as above using blood donated by an adult and the RT-PCR product pooled and purified using the QIAquick PCR Purification kit (QIAGEN). The DNA was then quantified using the nanodrop and FOXP3 copy numbers calculated using the Avogadro constant formula.

*Statistical analyses:* For paired comparisons between two time points random effects models were used to allow for the clustering effect of subject. For the antibody responses where there were 7 time points a generalised estimating equation was used with an exchangeable correlation structure. Responses were appropriately transformed and in the absence of a suitable transformation the data was ranked. All regressions were adjusted for possible confounding affects of sex, but due to well balanced groups there was very little evidence of confounding. Where appropriate, time and dose group interactions were tested. Significance was measured at the 5% level and all analyses were performed in Stata 11 (Statacorp) and figures drawn using Matlab 7.9 (The MathWorks Inc.).

## 3 Results

### 3.1 Recruitment and participation

The number of participants and their loss to the study at different time points are shown in Fig. 1. The overall refusal rate was 11.5%, loss to follow up due to the participant travelling was 17.4% and 3.8% of the children received an unscheduled measles vaccine.

### 3.2 Safety

The two dose regimen was safe since side effects were mild and infrequent. They did not differ in frequency or timing between group 1 and group 2 either at 4 months of age or at 9 months of age. The most frequent complaints were diarrhoea and fever with a mean prevalence of 7.9 ± 2.4% and 6.6 ± 2.7% respectively.

### 3.3 Measles and other antibody

**Fig. 2:**
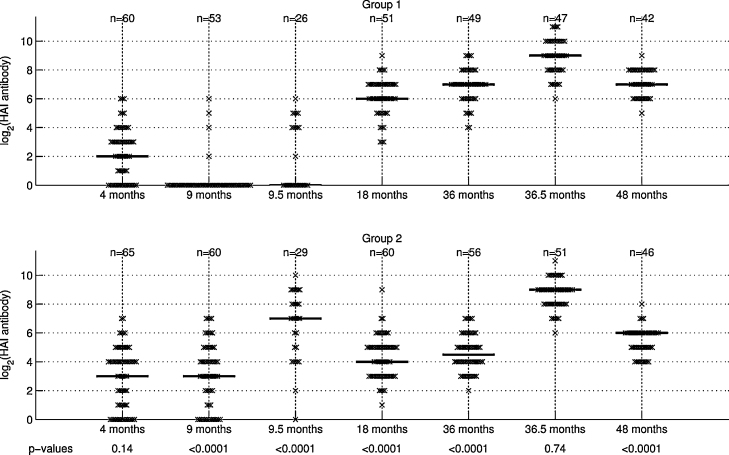
Measles haemaglutination-inhibiting antibody titres at various time points during the study. *p-*Values for differences between the groups are shown.

Before vaccination at 4 months of age median HAI titres were log_2_ 2 (IQR 0–3) and log_2_ 3 (IQR 1–4) in groups 1 and 2 respectively (Fig. 2 and Supplementary Table). At 9 months before the second measles vaccination the median HAI titre in group 2 was log_2_ 3 (IQR 1–6) which is significantly higher than that of group 1 which was zero; 77% of group 2 children had detectable antibody and 66% had protective levels whereas antibody was detected in only 6% of group 1 children. Two weeks after the second dose of E-Z vaccine antibody titres had risen sharply in group 2 with all but one child reaching protective levels whereas only 25/65 (36.4%) of group1 children attained these levels after their first measles vaccination.

At 18 months of age antibody titres in group 2 (median 4, IQR 3–5) fell significantly lower than those in group 1 (median 6, IQR 5–7) but then stabilised between 18 and 36 months. Both groups responded sharply to booster vaccination reaching equivalent and high concentrations (median titre 9, IQR 8–10). At 48 months of age antibody titres had dropped fourfold in group 1 (median 7, IQR 6–8) and eightfold in group 2 (median 6, IQR 5–6) although all subjects had protective levels of antibody. Responses did not vary significantly by sex.

In group 2 pre-vaccination antibody titres at 4 months were negatively and significantly correlated with titres at 9 and 18 months. Antibody titres at 18 and 36 months were positively and significantly correlated with those at 36 and 48 months respectively (Table 1).

Hepatitis B and Tetanus antibody measured at 18 months of age did not differ significantly between the two groups (data not shown).

### 3.4 Effector cell IFN-γ responses to measles or measles peptides

Table 2 shows the net number of IFN-γ ELI spots at different times of the study. At no time did the median numbers differ significantly between the groups nor was there a significant rise following a booster dose of the vaccine. However there was a significant fall in both groups between 36 and 48 months of age (*p* < 0.0001 in both cases).

Responses to pooled fusion peptides were low but rose significantly following the booster dose of measles vaccine at 36 months of age (*p* = 0.001 and *p* < 0.001 for group 1 and 2 respectively).

There was no significant correlation between antibody titres and effector responses to either virus or peptides at any time point (data not shown). Effector responses did not vary significantly by sex.

### 3.5 Measles-specific memory-cell responses

Table 3 shows the net IFN-γ ELIspot responses after 10 days of stimulation of PBMC with measles virus or pooled measles peptides. At 9 months of age responses of unvaccinated children (group 1) to pooled NP peptides were significantly lower than those in group 2 who had received E-Z vaccine at 4 months of age (*p* = 0.002). Thereafter there were no significant differences in cultured memory responses to the virus or peptides at 18 or 48 months of age. At no point did memory ELIspot responses correlate with measles antibody titres (data not shown) nor did they vary by sex.

### 3.6 Plasma cytokines

Levels of IL-10, lL-2Rα, IFN-γ and MIP-1β in plasma were measured before and two weeks after the booster dose of E-Z vaccine at 36 months of age (Table 4). In the case of IL-2, IL-5, IL-13 and IL-12 p40 levels were generally undetectable and data were not analysed. There were no significant differences between the groups at either of the time points nor did they vary by sex. The booster vaccination resulted in a significant fall in IL-10, IL-2Rα and MIP-1β levels in both groups (*p* < 0.001).

### 3.7 FOXP3 expression

There were no significant differences in FOX P3 expression (normalized against HUPO) between the groups or within the groups before or two weeks after the booster vaccination at 36 months of age. Before the boost median levels were 19.0 (IQR 3.7–39.0) and 23.6 (IQR 6.5–48.9) copies per mL for group 1 (*n* = 37) and group 2 (*n* = 39) subjects respectively. Two weeks afterwards median levels were 9.3 (IQR 2.8–26.6) and 20.4 (IQR 6.2–38.7) copies per ml for groups 1 and 2 respectively.

### 3.8 Flow cytometry for cytokine producing T-cells

Percentages of CD8 or CD4 T-cells expressing IFN-γ, CD69 or both markers in negative control cultures were subtracted from those in stimulated cultures. A net value of >0.1% was considered positive (Table 5).

*Memory cell assay at 9 months:* Only samples from group 2 infants were tested. In the majority of samples IFN-γ and CD69 responses to the nucleoprotein peptide pool were detectable in CD4 but not in CD8 T-cells.

*Effector cell assay at 9.5 months of age:* A similar but low proportion of CD4 and CD8 T-cells from the two groups showed a positive IFN-γ response after stimulation with E-D virus. There was concurrence of CD4 and CD8 IFN-γ responses in 6 of 7 samples. Expression of CD69 was detected more often in CD8 than CD4 T-cells.

*Memory cell assay at 18 months:* After stimulation with EZ virus IL-2 expression was detectable in less than half of the samples and very few expressed IFN-γ. There were no significant differences between cell types and little concurrence within the positive samples.

## 4 Discussion

Measles antibody protects against infection but its role in limiting viral multiplication and severity of disease is less clear [16]. Although an arbitrary protective level of measles antibody has been ascribed, in an outbreak of measles in Senegal half of the antibody negative vaccinated children did not develop measles when exposed [12]. In vaccinated macaques a rapid amnestic antibody response follows measles infection which coupled with a boost in cell mediated immunity limits viral replication and aborts disease [17]. With the assumption that a booster dose of vaccine mimics infection or exposure, we examined both antibody and cell mediated responses shortly after re-vaccination.

Our study is the first to provide detailed knowledge of the early antibody response to a booster dose of measles vaccine following either vaccine schedule. A standard dose of E-Z vaccine in 4 month old infants raised protective levels of antibody in the majority of the children by 9 months of age. After either one or two booster doses of vaccine antibody concentrations rose dramatically within 2 weeks and faded slowly with time. Maternal antibody, possibly by neutralising the live vaccine and altering antigen processing [18], depressed both primary and secondary antibody responses. The impact faded by 36 months of age and did not influence responses to further vaccination. The booster responses were independent of antibody at the time of vaccination suggesting that even if antibody concentrations are low a rapid response in conjunction with cellular immune responses will limit disease and lower transmission on subsequent measles exposure [19]. However concentrations of antibody following a boost decayed quicker in group 2 children. They may be more susceptible to subclinical infections [20] though this event is unlikely to result in the further spread of measles [21].

CD8 T-cells are necessary to control measles viraemia [16] and the role and importance of cytotoxic T-cell responses, cellular proliferative responses and cytokine responses during and after measles or primary vaccination have been thoroughly described [15,22,23]. However, very little is known of these responses shortly after booster vaccination or natural exposure in immunized children.

Early measles vaccination primed IFN-γ memory T-cell responses to nucleoprotein peptides which were significantly greater at 9 months of age in immunized than unimmunized infants. However some of the unimmunized infants in group 1 had responded to these peptides suggesting that common infections such as cytomegalovirus or Epstein-Barr virus prompt such responses [24]. At 18 and 48 months of age IFN-γ memory responses were readily detectable and similar in the two groups of children. Maternal antibody had no effect on these responses nor were they influenced by the number of times the child had been immunized.

Surprisingly ex vivo measles IFN-γ effector responses two weeks after vaccination did not differ between those receiving primary vaccination (group 1) or secondary vaccination (group 2). After a further boost at 36 months of age effector responses to E-Z virus were similar in both groups and in neither group was there a rise after the boost. However there was a small but significant rise to fusion peptides which did not differ between the groups.

Prime boost studies using recombinant Modified Vaccinia Ankara/TB vaccines in man [25] and DNA/measles vaccines in monkeys [17] indicate that maximum IFN-γ ELIspot responses occur 1–2 weeks after the booster immunization. Thus we are confident that the lack of a response after the booster doses was real and not due to late sampling. However macaques primed with DNA/measles protein vaccines raise cytotoxic T-cell, IFN-γ and antibody responses within 14 days of challenge with live virus [17,26]. Perhaps in our study the attenuated vaccine virus did not multiply sufficiently in the presence of antibody to raise a cell mediated immune response.

There were no significant differences in plasma cytokine levels between the groups before or after the 36 month booster dose which resulted in a significant fall in IL-10, IL-2Rα and MIP-1β concentrations in both groups after the boost. This was not mirrored by changes in FOXP3 mRNA expression which were expected to increase [27].

We found no relationship between maternal or vaccine derived measles antibody concentrations and IFN-γ ELIspot numbers or cytokine levels after primary or secondary immunization. Similar findings have been noted following primary measles immunization in infants [23] or after secondary immunization in children [28] or after measles in children [29].

Intracellular cytokine staining showed that CD4 and CD8 T-cells were equally prominent producers of IFN-γ during the effector response and that both cell types a produced IL-2 in memory responses. The memory response at 9 months of age following early vaccination consisted predominantly of CD4 T-cells. The finding fits with the idea that a Th-1 type response is predominant following vaccination [28] but contrasts with previous studies of cytotoxic T-cell activity during measles or after vaccination which reveal this response to be mainly due to CD8 T-cells [30]. Stimulation with 20-mer rather than shorter peptides may have favoured a CD4 T-cell response particularly in very young children.

Early two dose schedules of measles vaccine given at 6 and 9 months of age were recommended by WHO to control outbreaks and for use in countries with high attack rates of measles in infancy. Now WHO recommends such schedules in areas with a high incidence of HIV and measles [31]. However once measles is controlled in endemic areas the proportion of vaccinated mothers who have low levels of measles antibody will increase along with the proportion of unprotected infants. At present such children can only be protected by raising herd protection by supplemental measles vaccinations. Others have argued that if measles is to be eliminated and ultimately eradicated it would be better to strengthen routine services to achieve high coverage before deploying mass immunization [32,33]. An early two dose schedule would fit well into this scheme: it protects the very young [5] and the HIV infected [34], increases coverage [4] and enhances child survival [6]. Additional doses could be given if outbreaks occur or if measles is to be eliminated or eradicated.

## Figures and Tables

**Table 1:** Correlation of pre and post vaccination HAI titres in group 2.

Age (months)		9	9.5	18	36	36.5	48
4	*r*	−0.48	−0.45	−0.40	−0.06	0.20	−0.18
	*p*	**0.0002**	0.021	**0.0023**	0.65	0.18	0.25
	*n*	56	26	56	52	47	42

9	*r*		0.48	0.29	0.24	0.19	0.40
	*p*		0.01	0.04	0.09	0.22	**0.0085**
	*n*		26	53	49	45	42

9.5	*r*			0.26	−0.01	0.28	−0.04
	*p*			0.19	0.97	0.17	0.87
	*n*			27	27	25	23

18	*r*				0.67	−0.18	0.56
	*p*				**<0.0001**	0.217	**0.0001**
	*n*				54	49	45

36	*r*					−0.0003	0.63
	*p*					0.99	**<0.0001**
	*n*					51	46

36.5	*r*						0.03
	*p*						0.84
	*n*						44

*r*, spearman correlation; *p*, *p*-value; *n*, number of subject pairs. Highlighted *p*-values are those significant after correction for multiplicity.

**Table 2:** IFN-γ ELIspot effector responses (expressed as net SFU per 10^6^ PBMC) in the two groups at various ages.

Age (months)	Stimulus	Group 1	Group 2
		Median (1QR) *n*	Median (1QR) *n*
9.5	E-D virus	85 (25–310) 24	35 (10-130) 30
36	E-Z virus	145 (90–255) 48	130 (73–275) 55
36.5	E-Z virus	150 (160–220) 46	120 (70–250) 46
48	E-Z virus	20 (10–30) 43	20 (10–40) 44
36	Fusion peptides	0 (0–10) 51	0 (0–20) 53
36.5	Fusion peptides	10 (10–80) 47	10 (0–50) 46
48	Fusion peptides	10 (10–20) 43	10 (0–20) 45

**Table 3:** IFN-γ memory responses (expressed as net SFU per 10^6^ PBMC) in the two groups at various times of the study.

Age (months)	Stimulus	Group 1	Group 2
		Median (1QR) *n*	Median (1QR) *n*
9	NP peptides	80 (10–600) 20	540 (150–1475) 29
18	E-Z virus	1260 (545–2145) 27	1395 (510–1915) 32
18	NP peptides	740 (175–1775) 32	260 (58–1115) 37
48	E-Z virus	500 (65–1437) 35	810 (365–1518) 33
48	Fusion peptide	870 (332–1402) 35	1045 (535–2150) 32

**Table 4:** Cytokine levels (pg/mL) pre and post a booster dose of E-Z measles vaccine at 36 months of age.

Cytokine	Time	Group	Median	Lower quartile	Upper quartile	*n*
IFNγ	Pre	1	60.2	0	102.0	50
	Post	1	26.1	0	63.1	50
	Pre	2	31.8	0	172.6	55
	Post	2	38.1	0	85.8	55

IL-10	Pre	1	0.9	0.2	2.0	50
	Post	1	0	0	0.8	50
	Pre	2	0.7	0	1.7	55
	Post	2	0	0	0.5	55

IL-2Rα	Pre	1	207.5	94.8	331.3	50
	Post	1	64.2	38.5	115.6	50
	Pre	2	175.1	0	380.7	55
	Post	2	55.2	21.8	85.5	55

MIP-1β	Pre	1	10.7	4.5	18.2	50
	Post	1	3.7	0	11.3	50
	Pre	2	10.8	0	21.6	55
	Post	2	5.1	0	12.0	55

**Table 5:** Numbers and percentages of individuals with responses to measles detectable by flow cytometry. Statistics based on comparison between expression of markers by CD4 and CD8 T-cells, based on Fisher's exact probability test.

Age (months)	Assay	Received 4 month vaccine	Markers	CD8 T-cells			CD4 T-cells			*p*
				Undetectable	Detectable	% detectable	Undetectable	Detectable	% detectable	
9	Memory response 10 days measles peptide	Yes	IFNγ	9	2	18	1	10	91	0.002
			CD69	9	2	18	2	9	82	0.009
			CD69 + IFNγ	9	2	18	4	7	64	NS

9.5	Effector response 18 h E-D measles virus stimulation	Yes	IFNγ	14	4	22	14	4	22	NS
			CD69	12	6	33	17	1	6	NS
			CD69 + IFNγ	16	2	11	17	1	6	NS
		No	IFNγ	13	3	19	13	3	19	NS
			CD69	13	3	19	14	2	13	NS
			CD69 + IFNγ	14	2	13	15	1	6	NS
		Combined	IFNγ	27	7	21	27	7	21	NS
			CD69	25	9	26	31	3	9	NS
			CD69 + IFNγ	30	4	12	32	2	6	NS

18	Memory response 10 days E-Z measles virus stimulation	Yes	IFNγ	6	1	14	6	1	14	NS
			IL-2	5	2	29	6	1	14	NS
			IFNγ + IL-2	7	0	0	7	0	0	NS
		No	IFNγ	8	0	0	7	1	13	NS
			IL-2	5	3	38	5	3	38	NS
			IFNγ + IL-2	8	0	0	8	0	0	NS
		Combined	IFNγ	14	1	7	13	2	13	NS
			IL-2	10	5	33	11	4	27	NS
			IFNγ + IL-2	15	0	0	15	0	0	NS
