# Supplementary material for: Immunological impact of an additional early measles vaccine in Gambian children: Responses to a boost at 3 years
Source: Vaccine. 2012 Mar 28;30(15):2543–50. doi: 10.1016/j.vaccine.2012.01.083 (PMC3401374; doi:10.1016/j.vaccine.2012.01.083)
Supplement: Supplementary file 1 [file mmc1.doc]

**Supplementary table**

Median (IQR) antibody titres and proportion of children at different ages with non protective titres (< log_2_ 3 ) or with no detected antibody.

| age | **1 dose** | **2 dose** | **p-value for 2 dose vs. 1 dose** |
| --- | --- | --- | --- |
| 4 months median (IQR) | 2 (0 to 3)  53.33  28.33 | 3 (1 to 4)  41.54  23.08 | 0.014 |
| % less than 3 |
| % equal to zero |
| 9 months | 0 ( 0 to 0)  94.34  92.45 | 3 ( 1 to 6)  36.67  23.33 | <0.0001 |
|  |
|  |
| 9.5 months | 0 (0 to 4)  61.54  57.69 | 7 (4.75 to 8.25)  6.90  3.45 | <0.0001 |
|  |
|  |
| 18 months | 6 (5.25 to 7)  0  0 | 4 ( 3 to 5)  8.33  0 | <0.0001 |
|  |
|  |
| 36 months | 7 (6 to 8)  0  0 | 4.5 (4 to 5.5)  1.79  0 | <0.0001 |
|  |
|  |
| 36.5 months | 9 (8 to 10)  0  0 | 9 (8 to 9)  0  0 | 0.74 |
|  |
|  |
| 48 months | 7 (6 to 8)  0  0 | 6 ( 5 to 6)  0  0 | <0.0001 |
|  |
|  |
